# The association between vascular endothelial growth factor gene polymorphisms and stroke: a meta‐analysis

**DOI:** 10.1002/brb3.482

**Published:** 2016-05-03

**Authors:** Tao Wu, Shi Qiu, Peifu Wang, Jilai Li, Qin Li, Jichen Du

**Affiliations:** ^1^ Department of NeurologyAerospace Center HospitalBeijingChina

**Keywords:** Meta‐analysis, polymorphism, stroke, vascular endothelial growth factor

## Abstract

**Objectives:**

Numerous studies have investigated the relationships between vascular endothelial growth factor (VEGF) gene polymorphisms and stroke. However, their findings remain controversial. The objective of this study was to evaluate the relationships between VEGF gene polymorphisms and stroke by a meta‐analysis.

**Materials and methods:**

The PubMed, Embase, China National Knowledge Infrastructure database, Wanfang Chinese database, and VIP Chinese database were systemically searched. Data were extracted by two independent reviewers, and pooled odds ratio (OR) with 95% confidence interval (CI) were calculated.

**Results:**

Ten studies were included, including a total of 2331 cases and 1814 controls for +936C>T, 3040 cases and 2649 controls for −1154G>A. Under the dominant and recessive models, respectively, the overall ORs and 95% CIs of +936 T were 1.44, 1.09–1.90, *P *=* *0.01 (1.53, 1.14–2.05, *P *=* *0.005, in Asians) and 1.19, 0.85–1.65, *P *=* *0.31, and the overall ORs and 95% CIs of −1154 A were 0.98, 0.87–1.10, *P *=* *0.75 and 0.95, 0.82–1.11, *P *=* *0.53. No publication bias was found in this meta‐analysis.

**Conclusions:**

The meta‐analysis showed that +936C>T may be a risk factor for stroke, especially in Asians, while −1154G>A was not associated with stroke.

## Introduction

Stroke is one of the most common causes of death and the most common cause of long‐term disability in the world, which endangers human health and increased the burden on society (Traylor et al. 2012). Researchers have established that stroke is a heterogeneous multifactorial disease caused by both environmental and genetic factors (Bevan et al. 2012). Environmental factors, such as tobacco smoking, high blood pressure, diabetes, and obesity may contribute to the development of stroke (Hankey 2006). On the other hand, numerous genetic association studies have been performed to find the possible gene polymorphisms associated with stroke.

Vascular endothelial growth factor (VEGF) is a prime angiogenic factor and a regulator of endothelial cell proliferation. The VEGF gene is located on chromosome 6p21, which is comprised of a 14 kb coding region with 7 introns and 8 exons (Vincenti et al. 1996). A number of studies have reported that there are at least 30 single‐nucleotide polymorphisms (SNPs) in VEGF gene. However, only about three or four polymorphisms could affect the VEGF expression (Brogan et al. 1999; Lambrechts et al. 2003). So far, two of them were investigated most frequently in the included studies, which may play a critical role in the mechanism of stroke: +936C>T (rs3025039), and −1154G>A (rs1570360). −1154G>A is located in the promoter region, which is related to the translation start site and associated with decreased VEGF expression (Brogan et al. 1999; Watson et al. 2000), and +936C>T polymorphism is in the 3′‐untranslated region which is associated with decreased serum VEGF levels (Renner et al. 2000).

A growing number of studies have been conducted to assess the relationship of VEGF gene polymorphisms with stroke. Although numerous studies have reported the association between these gene polymorphisms and stroke, the findings still remain controversial. Therefore, we performed this meta‐analysis to observe these correlations.

## Methods

### Search strategy

We carried out a publication search for the potential eligible articles in English and Chinese in the following databases: (1) Medline in PubMed searching engine; (2) Embase database; (3) Chinese National Knowledge Infrastructure (CNKI) database; (4) Wanfang Chinese database; and (5) VIP database. The latest date for searching literatures was November 1st, 2015. The key words for literature searching were: [“vascular endothelial growth factors” or “vasculotropin” or “VEGF”] and [“stroke” or “cerebral infarction” or “cerebrovascular disorders”] and [“single nucleotide polymorphism” or “SNP” or “polymorphism” or “mutation” or “genetics” or “variant”]. Publication language was restricted to English and Chinese, and the subjects were not limited in our search. We also performed a manual search of the reference lists of retrieved articles to find additional potential studies.

### Inclusion criteria

The inclusion criteria for the gene association studies in this meta‐analysis were as follows: (1) independently published studies explored the association between VEGF gene polymorphisms and stroke; (2) with genotype or allelic distributions provided; (3) with data in any of the two polymorphisms, and sufficient data available to calculate an odd ratio (OR) with its 95% confidential interval (CI); (4) if the authors published two or more studies using the same subjects, the most recent publication or the publication with the largest sample size was included. No limitations were placed on race, ethnicity, or geography area.

### Data extraction

Relevant data were systematically extracted from the included studies by two authors using a standardized form, and reached a consensus on all items. The researchers collected the following data: the first author's name, publication year, countries and ethnicities of participants, sample size, and genotyping method.

### Quality score assessment

To determine the methodological quality of the included studies, we used the Newcastle–Ottawa scale (NOS) (Wells et al. 2011) to judge the quality of these case–control studies. The NOS ranges from zero to nine stars, and a score ≥7 was considered to be of high quality. Two authors assessed the quality of included studies independently, and all disagreements were resolved by discussion.

### Evaluation of statistic association

We performed the association between +936C>T and −1154G>A and the risk of stroke by calculating OR and 95% CI. We estimated the recessive model and dominant model for genotype comparison. Pooled ORs were using the method of Mantel–Haenszel or DerSimonian–Laird, and 95% CI was estimated by Woolf's method. Hardy–Weinberg equilibrium (HWE) of the distribution of controls was checked by Pearson's *χ* ^2^ test. Heterogeneity between studies was estimated by Cochran's *χ* ^2^ based *Q*‐statistic test (Colditz et al. 1995) and *I* ^2^ test. The heterogeneity was considered to be statistically significant at *P *≤* *0.1 or *I* ^2^ > 50%. When the *P* value was >0.1 and *I* ^2^ ≤ 50%, the pooled OR was calculated by fixed‐effects model, otherwise, the random effects was applied. Sensitivity analysis of the summary OR coefficients is computed by omitting each study in turn. We also performed subgroup analysis and meta‐regression to investigate potential sources of heterogeneity. Publication bias was explored using funnel plots and Egger's regression test (*P *<* *0.05 indicated statistical significance) (Egger et al. 1997). All statistic tests were conducted by RevMan 5.2 (The Nordic Cochrane Centre, Copenhagen, Denmark) and Stata 11.0 (Stata Corporation, College Station, TX).

## Results

### Included studies

**Figure 1:**
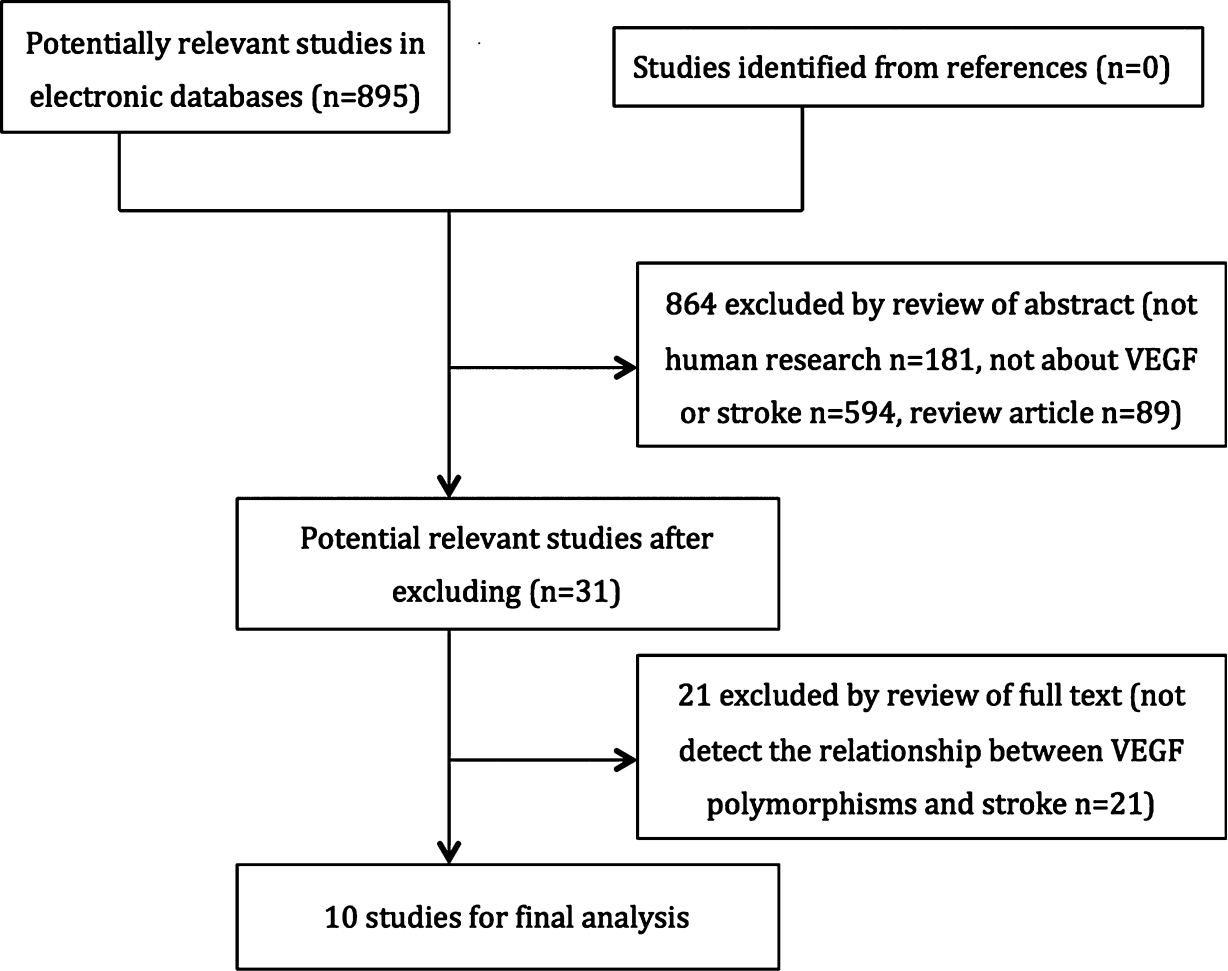
Flowchart of the literature search and selection.

Figure 1 showed the process of retrieving eligible studies. Initially, our highly sensitive search strategy identified 895 articles. After reviewed the titles and abstracts of all articles, 864 articles were excluded. After systematically reading full texts, we excluded another 21 articles. Finally, 10 case–control studies with a total of 4233 patients with stroke and 3838 control subjects met our inclusion criteria for qualitative data analysis (Rueda et al. 2005; Li and Jin 2007; Liu et al. 2007; Zhang et al. 2007, 2014; Li et al. 2009; Fu et al. 2011; Kim et al. 2011; Yu et al. 2012; Fontanella et al. 2013). There was no genome‐wide association study (GWAS) relevant to VEGF gene variants and stroke. No population overlapping and duplicate publication existed in the included studies. Table 1 summarized the characteristics of the studies included in the meta‐analysis. Eight case–control studies with 2331 cases and 1814 controls for +936C>T, four studies with 3040 cases and 2649 controls for −1154G>A were selected eventually. Table 2 showed the studies that have provided the distribution of VEGF genotype and allele among stroke patients and controls.

**Table 1:** Characteristics of studies included in the meta‐analysis

Author	Year	Country	Ethnicity	Age (year)	Male (%)	Sample size		Genotype method	Polymorphism	Newcastle–Ottawa scale score
						Case	Control			
Rueda et al.	2005	Spain	European	74.5 ± 6.0	42.7	53	226	TaqMan	−1154G/A	7
Liu et al.	2007	China	Asian	67.0 ± 9.8	58.7	155	195	PCR‐RFLP	+936C/T	9
Zhang et al.	2007	China	Asian	60.3 ± 9.4	62.7	1849	1798	PCR‐RFLP	−1154G/A	9
Li et al.	2007	China	Asian	62.0 ± 10.3	53.5	200	100	PCR‐RFLP	+936C/T	9
Li et al.	2009	China	Asian	66.2 ± 5.0	48.0	150	120	PCR‐RFLP	+936C/T	7
Kim et al.	2011	South Korea	Asian	63.5 ± 11.4	56.6	991	494	PCR	+936C/T,−1154G/A	9
Fu et al.	2011	China	Asian	64.8 ± 9.6	57.1	147	131	PCR‐RFLP	+936C/T,−1154G/A	8
Yu et al.	2012	China	Asian	65.4 ± 8.2	67.7	420	456	PCR‐RFLP	+936C/T	7
Fontanella et al.	2013	Italy	European	55.3 ± 12.0	34.5	200	200	PCR	+936C/T	8
Zhang et al.	2014	China	Asian	57.6 ± 10.1	51.2	68	118	PCR‐RFLP	+936C/T	6

**Table 2:** Distribution of vascular endothelial growth factor genotype and allele among stroke patients and controls in two single‐nucleotide polymorphisms

Author	Sample size		+936C/T						−1154G/A					
			C	T	CC	CT	TT	HWE	G	A	GG	GA	AA	HWE
Rueda et al. (2005)	Case	53	–	–	–	–	–		77	29	26	25	2	
	Control	226	–	–	–	–	–		320	132	118	84	24	0.13
Liu et al. (2007)	Case	155			90				–	–	–	–	–	
	Control	195			150				–	–	–	–	–	
Zhang et al. (2007)	Case	1849	–	–	–	–	–		2018	1680	539	940	370	
	Control	1798	–	–	–	–	–		1937	1659	515	907	376	0.00
Li et al. (2007)	Case	200	285	115	125	35	40		–	–	–	–	–	
	Control	100	154	46	70	14	16	0.00	–	–	–	–	–	
Li et al. (2009)	Case	150	190	110	51	88	11		–	–	–	–	–	
	Control	120	180	60	67	46	7	0.80	–	–	–	–	–	
Kim et al. (2011)	Case	991	1604	378	642	320	29		1619	363	674	271	46	
	Control	494	824	164	344	136	14	0.89	815	173	339	137	18	0.37
Fu et al. (2011)	Case	147	249	45	106	37	4		227	67	86	55	6	
	Control	131	218	44	90	38	3	0.66	194	68	69	56	6	0.20
Yu et al. (2012)	Case	420	573	267	172	229	19		–	‐	–	–	–	
	Control	456	706	206	267	172	17	0.09	–	–	–	–	–	
Fontanella et al. (2013)	Case	200	350	50	153	44	3		–	–	–	–	–	
	Control	200	348	52	151	46	3	0.81	–	–	–	–	–	
Zhang et al. (2014)	Case	68	114	22	48	18	2		–	–	–	–	–	
	Control	118	196	40	81	34	3	0.80	–	–	–	–	–	

### Association of VEGF gene polymorphisms and stroke susceptibility

**Figure 2:**
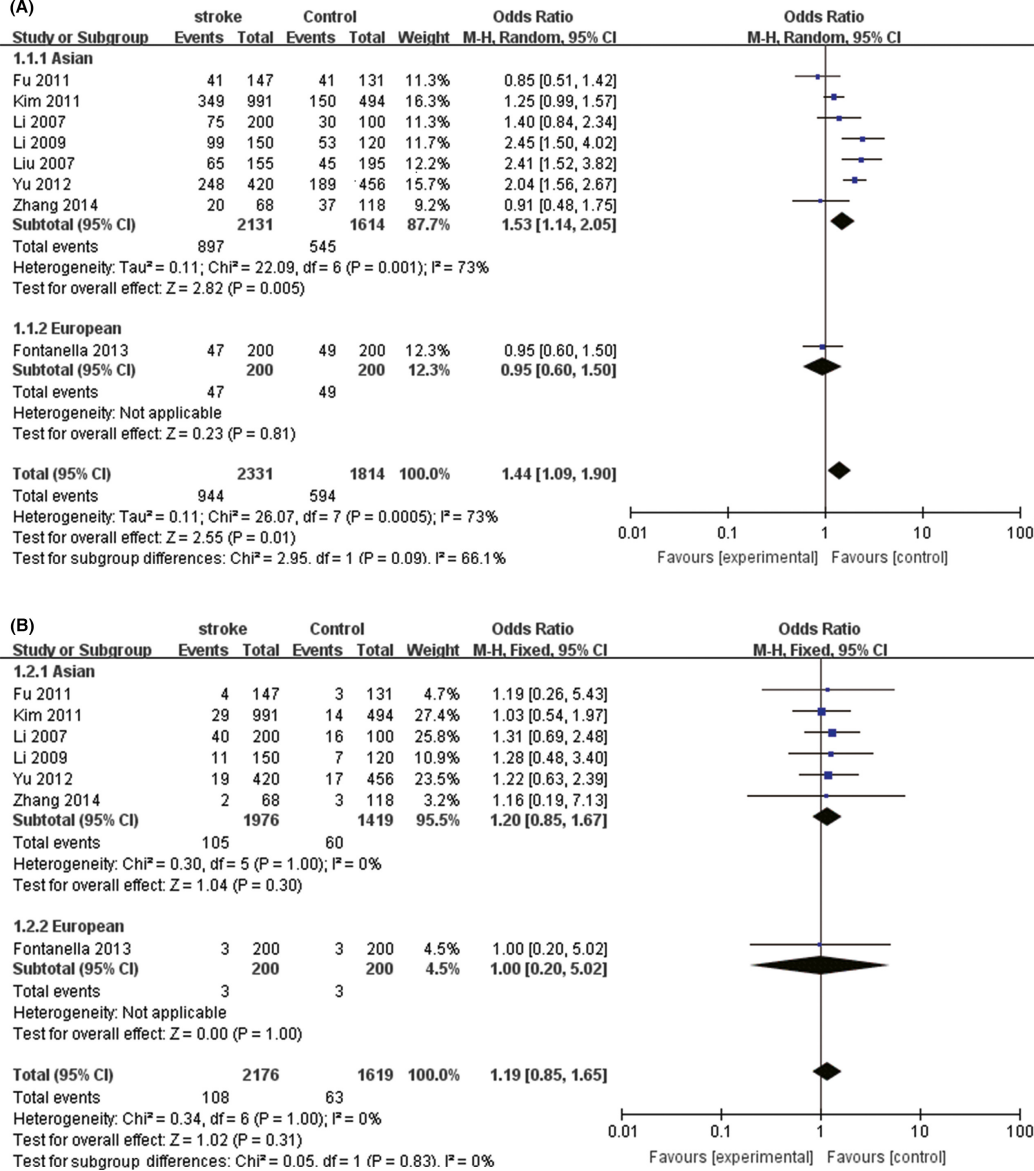
The association between +936C/T and stroke in different genetic models. (A) Dominant model. (B) Recessive model.

**Figure 3:**
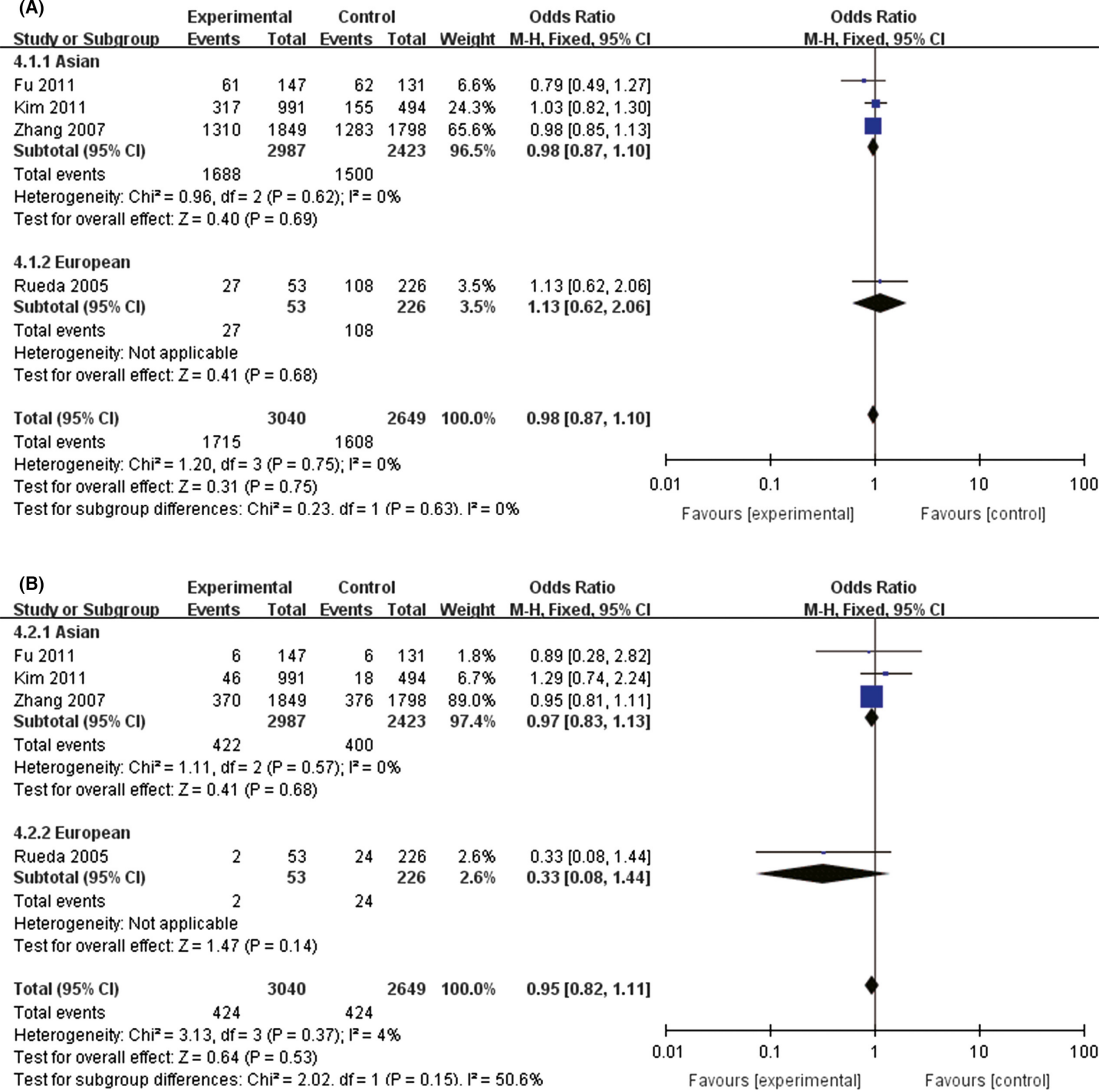
The association between −1154G>A and stroke in different genetic models. (A) Dominant model. (B) Recessive model.

We compared the minor allele and major allele in the dominant and recessive models. The overall ORs and 95% CIs of +936 T were 1.44, 1.09–1.90 (*P *=* *0.01) and 1.19, 0.85–1.65 (*P *=* *0.31) compared with C in the dominant and recessive models, respectively (Fig. 2), of which dominant model was more significant in Asians (OR = 1.53, 95% CI = 1.14–2.05, *P *=* *0.001). The overall ORs and 95% CIs of −1154 A were 0.98, 0.87–1.10 (*P *=* *0.75) and 0.95, 0.82–1.11 (*P *=* *0.53) compared with G in the dominant and recessive models, respectively (Fig. 3) (Table 3).

**Table 3:** The association between vascular endothelial growth factor gene polymorphisms and stroke in different genetic models

Gene polymorphism	Number of studies	Genetic model	Odds ratio	95% CI	*P* value
+936C>T	8	Dominant	1.44	1.09–1.90	0.01
		Recessive	1.19	0.85–1.65	0.31
−1154G>A	4	Dominant	0.98	0.87–1.10	0.75
		Recessive	0.95	0.82–1.11	0.53

### Sensitivity analysis

**Figure 4:**
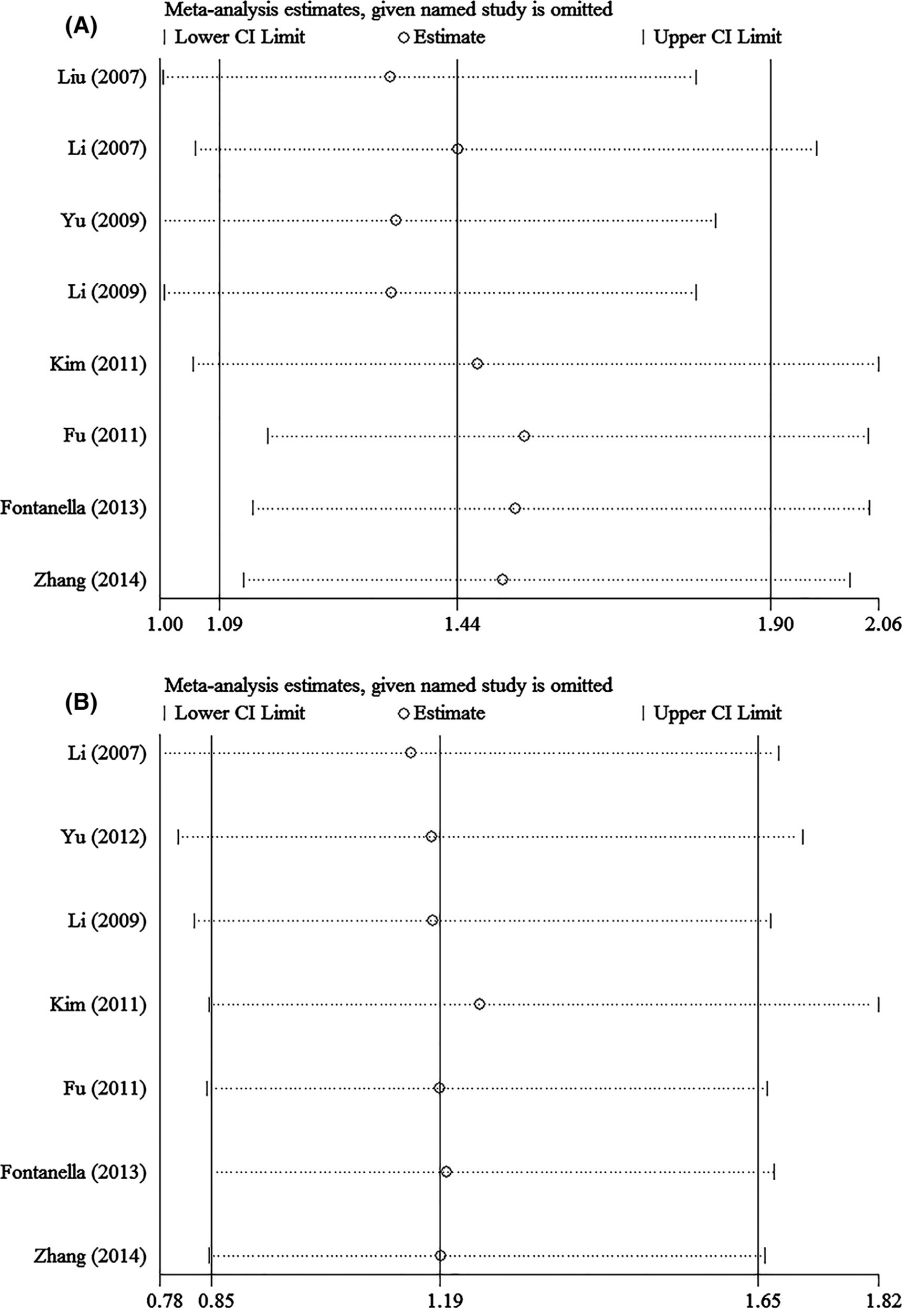
Sensitivity analysis in assessing publication bias about +936 C/T and stroke in different genetic models. (A) Dominant model. (B) Recessive model.

**Figure 5:**
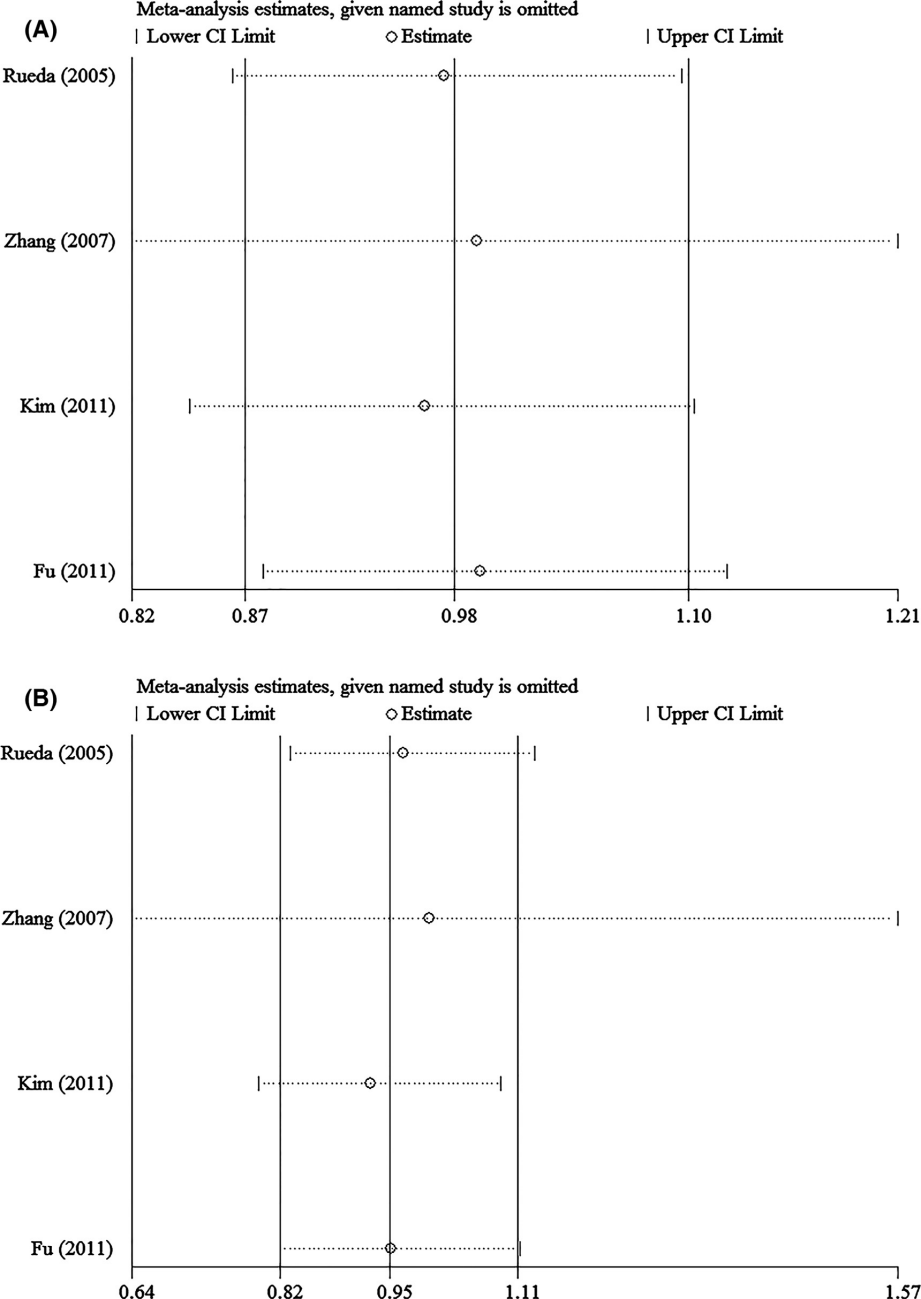
Sensitivity analysis in assessing publication bias about −1154G>A and stroke in different genetic models. (A) Dominant model. (B) Recessive model.

Sensitivity analysis was performed to assess the stability of results. Sensitivity analysis of the summary OR coefficients on the relationships of the two SNPs and the risk of stroke is computed by omitting each study in turn. However, the corresponding ORs were not substantially altered in comparisons, indicating that our results were relatively robust. The results of sensitivity analysis were shown in Figures 4 and 5.

### Heterogeneity and publication bias

There was no heterogeneity in recessive model of +936C>T and both recessive and dominant model of −1154G>A (*I* ^2^ = 0%, 0%, 4%, respectively), except a relatively high heterogeneity in dominant model of +936C>T (*I* ^2^ = 73%). Given this we performed subgroup analysis and meta‐regression to investigate the sources of heterogeneity. Age, gender, ethnicity, sample size, genotyping method, publication language were tested, the results confirmed the sources to be none of them (Table 4).

**Table 4:** Meta‐regression analysis of potential source of heterogeneity

Factors	Coefficient	SE	*t*	*P* value	95% CI	
					LL	UL
Age	0.111	0.040	2.77	0.221	−0.398	0.619
Gender	−0.012	0.040	−0.29	0.818	−0.514	0.490
Ethnicity	0.622	0.520	1.20	0.443	−5.983	7.226
Sample size	0.000	0.001	0.22	0.865	−0.013	0.014
Genotype method	0.249	1.327	0.19	0.882	−16.611	17.109
Language	−0.796	0.343	−2.32	0.259	−5.15	3.56

SE, standard error; UL, upper limit; LL, lower limit.

**Figure 6:**
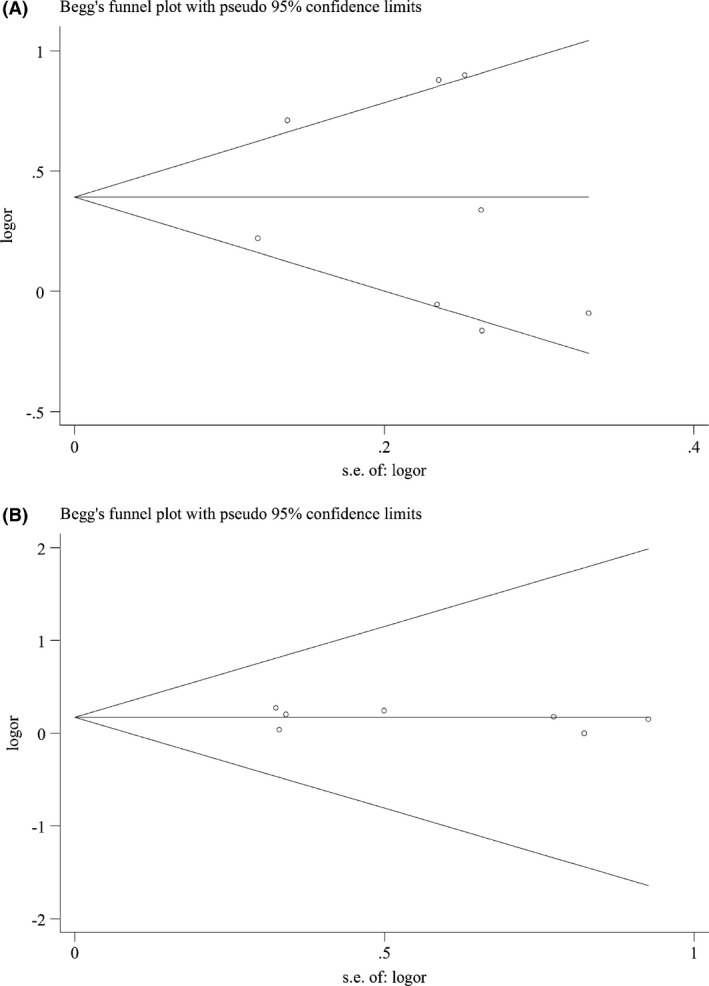
Egger's funnel plot in assessing publication bias about +936 C/T and stroke in different genetic models. (A) Dominant model. (B) Recessive model.

Funnel plot and Egger's linear regression test were performed to detect publication bias. The shape of funnel plot seemed symmetrical for all comparison models, and the Egger's test was used to provide statistical evidence of publication funnel plot symmetry. The result also did not reveal any evidence of publication bias (*P* = 0.73, 0.75, 0.89, 0.74, respectively) (Fig. 6) (Table 5).

**Table 5:** Egger's linear regression test to measure the funnel plot asymmetric

Polymorphism	Comparisons	Study	*t*	*P* value	95% CI
+936C>T	Dominant	Overall	−0.37	0.73	−5.90 to 4.36
	Recessive	Overall	−0.33	0.75	−0.76 to 0.59
−1154G>A	Dominant	Overall	−0.16	0.89	−3.51 to 3.27
	Recessive	Overall	−0.38	0.74	−4.13 to 3.46

## Discussion

Angiogenesis have been found to be critical to the progression of stroke. VEGF is an essential vascular growth factor, and the expression level of VEGF has been suggested to be associated with the increased risk of stroke (Greenberg and Jin 2013). The reason may be relevant to the diverse angiogenic functions of VEGF. VEGF plays a role in the progression of atherogenesis, plaque instability, and collateral vessel development in stroke. It could act on vasa vasorum of atherosclerotic arteries to promote angiogenesis, which may lead to intraplaque hemorrhage and plaque rupture in some cases (Sluimer and Daemen 2009). On the other hand, VEGF has the ability to increase vascular permeability and cause vasodilatation, which may be associated with hemorrhage disorders (Jung et al. 2006). Hence, the increased level of VEGF has a broad array of effects related to stroke and lead to a predisposition to stroke.

The VEGF gene includes two relatively most frequently investigated SNPs, +936C>T (rs3025039) and −1154G>A (rs1570360), which are all established to influence VEGF expression. The relationship between two polymorphisms and stroke remains obscure due to inconsistent results. To our knowledge, up to now, no such meta‐analysis has been performed to investigate the relationship between VEGF gene polymorphisms and the risk of stroke. Whether the two polymorphisms can affect the risk of stroke is concerned in this study particularly.

In the present meta‐analysis, we investigated the relationship between two VEGF gene polymorphisms and stroke susceptibility. Data extracted from published studies were combined to estimate genetic associations between the most commonly investigated polymorphisms of VEGF, +936C>T and −1154G>A, and stroke. The results of the meta‐analysis indicated that one of the two polymorphisms, +936C>T, may be associated with the increased risk of stroke. Individuals with +936 T showed increased risk of stroke in dominant model (OR = 1.44, 95% CI = 1.09–1.90, *P *=* *0.01), which was more significant in Asians (OR = 1.53, 95% CI = 1.14–2.05, *P *=* *0.001). However, the data indicated no relationship between −1154G>A and stroke, in both models.

Sensitivity analysis of the summary OR coefficients on the relationships of the two SNPs and the risk of stroke is computed by omitting each study in turn. As a result, the corresponding ORs were not substantially altered in comparisons, indicating that our results were relatively robust. It is worth noting that the combined OR in analysis of −1154G>A could be influenced by a certain study with the most large sample size, which indicated that more well‐designed studies with large sample size were required.

There was no heterogeneity in recessive model of +936C>T and both the recessive and dominant model of −1154G>A, except a relatively high heterogeneity in dominant model of +936C>T. Given this we performed subgroup analysis and meta‐regression to investigate the sources of heterogeneity. However, the results indicated that the source of heterogeneity was neither age, gender, ethnicity, sample size, genotyping method, nor publication language. In order to reduce the effect of the heterogeneity to the most extent, we used the random effects model to calculate the pooled OR, and the result remained positive. In the meanwhile, the shape of funnel plot seemed to be symmetrical for all comparison models and the result of Egger's test showed there was no publication bias in this meta‐analysis.

It remains debatable how the VEGF signaling pathway affect the pathogenesis of stroke. Evidences support the involvement of angiogenesis in stroke. VEGF have important roles in the development and function of the circulation system, which have been shown to participate in atherosclerosis and angiogenesis (Greenberg and Jin 2013). On the one hand, several studies suggest that increased VEGF signaling aggravates atherosclerosis through neovascularization and inflammation in atheromatous plaques. Increased density of microvessels within the plaque contributes to the growth and destabilization of the plaque, resulting in the narrowing and occlusion of large cerebral arteries (Celletti et al. 2001; Moulton 2006). On the other hand, the lack of sufficient VEGF signaling could result in endothelial dysfunction, vascular degeneration, and formation of weak, thin‐walled vasculature, which can reduce vessel compliance and increase the risk of spontaneous vessel wall rupture (Qureshi et al. 2001; Greenberg and Jin 2005). To investigate whether the polymorphisms of VEGF are associated with the risk of stroke, may contribute to the study of the mechanisms of stroke.

The pathogenesis of stroke is complex and genetic factors play an important role in stroke susceptibility. The relationship between genes and stroke has been confirmed in other meta‐analysis, such as methylenetetrahydrofolate reductase (MTHFR), IL‐1 and matrix metalloproteinases (MMP) (Li and Qin 2014; Wen et al. 2014; Zou et al. 2015). These meta‐analysis revealed that genetic mutations were significant in stroke. More studies are required to detect the relationship of genes and stroke. Our meta‐analysis did not reveal any evidence of association between −1154G>A and stroke, but the results suggested that +936C>T may be associated with the risk of stroke.

The current meta‐analysis has limitations that should be acknowledged. First, the number of studies enrolled in this meta‐analysis was relatively small. Some null study findings may be not published. Second, all included articles were published in English or Chinese. Therefore, studies issued in other languages might be missed. Third, there was heterogeneity in the dominant model of +936C>T, and we failed to find the source of the heterogeneity by subgroup analysis and meta‐regression. Even though we used the random effects model to reduce its affect, it still makes the results uncertain. Fourth, as the number of included studies was relatively small and some of the original articles didn't provide more details of the subtypes, we failed to perform stroke subtypes analysis. In addition, we analyzed five genetic models, allele model, dominant model, recessive model, additive model, and codominant model, there were significant findings only in the dominant model.

## Conclusions

In conclusion, our results suggested that the +936C>T polymorphisms may be associated with the risk of stroke, especially in Asians, but not −1154G>A polymorphisms. And more studies in this field are still needed to make the association more conclusive.

## Conflict of Interest

The authors report no conflicts of interest. The authors alone are responsible for the content and writing of the paper.
